# Reaching the last mile: main challenges relating to and recommendations to accelerate onchocerciasis elimination in Africa

**DOI:** 10.1186/s40249-019-0567-z

**Published:** 2019-07-04

**Authors:** Gebremedhin Gebrezgabiher, Zeleke Mekonnen, Delenasaw Yewhalaw, Asrat Hailu

**Affiliations:** 1 School of Medical Laboratory Sciences, Institute of Health Sciences, Jimma University, P.O. Box 378, Jimma, Ethiopia; 2 College of Veterinary Medicine, Samara University, Samara, Ethiopia; 3 Tropical and Infectious Diseases Research Center, Jimma University, Jimma, Ethiopia; 4 Department of Microbiology, Immunology, and Parasitology, School of Medicine, College of Health Sciences, Addis Ababa University, Addis Ababa, Ethiopia

**Keywords:** Onchocerciasis, Elimination, Challenge, Africa

## Abstract

**Background:**

Onchocerciasis (river blindness), caused by the filarial worm species *Onchocerca volvulus*, is a serious vector-borne neglected tropical disease (NTD) of public health and socioeconomic concern. It is transmitted through the bite of black flies of the genus *Simulium*, and manifested in dermal and ocular lesions. Ninety-nine percent of the total global risk and burden of onchocerciasis is in Africa. This scoping review examines the key challenges related to the elimination of onchocerciasis by 2020–2025 in Africa, and proposes recommendations to overcome the challenges and accelerate disease elimination. To find relevant articles published in peer-reviewed journals, a search of PubMed and Google Scholar databases was carried out.

**Main text:**

Rigorous regional interventions carried out to control and eliminate onchocerciasis in the past four decades in Africa have been effective in bringing the disease burden under control; it is currently not a public health problem in most endemic areas. Notably, transmission of the parasite is interrupted in some hyperendemic localities. Recently, there has been a policy shift from control to complete disease elimination by 2020 in selected countries and by 2025 in the majority of endemic African countries. The WHO has published guidelines for stopping mass drug administration (MDA) and verifying the interruption of transmission and elimination of human onchocerciasis. Therefore, countries have revised their plans, established a goal of disease elimination in line with an evidence based decision to stop MDA and verify elimination, and incorporated it into their NTDs national master plans.

Nevertheless, challenges remain pertaining to the elimination of onchocerciasis in Africa. The challenge we review in this paper are: incomplete elimination mapping of all transmission zones, co-endemicity of onchocerciasis and loiasis, possible emergence of ivermectin resistance, uncoordinated cross-border elimination efforts, conflict and civil unrest, suboptimal program implementation, and technical and financial challenges. This paper also proposes recommendations to overcome the challenges and accelerate disease elimination. These are: a need for complete disease elimination mapping, a need for collaborative elimination activities between national programs, a need for a different drug distribution approach in conflict-affected areas, a need for routine monitoring and evaluation of MDA programs, a need for implementing alternative treatment strategies (ATSs) in areas with elimination anticipated beyond 2025, and a need for strong partnerships and continued funding.

**Conclusions:**

National programs need to regularly monitor and evaluate the performance and progress of their interventions, while envisaging the complete elimination of onchocerciasis from their territory. Factors hindering the targeted goal of interruption of parasite transmission need to be identified and remedial actions should be taken. If possible and appropriate, ATSs need to be implemented to accelerate disease elimination by 2025.

**Electronic supplementary material:**

The online version of this article (10.1186/s40249-019-0567-z) contains supplementary material, which is available to authorized users.

## Multilingual abstracts

Please see Additional file 1 for translations of the abstract into the five official working languages of the United Nations.

## Background

Onchocerciasis, also called river blindness, is a vector-borne parasitic neglected tropical disease (NTD) caused by the filarial nematode *Onchocerca volvulus*. It is transmitted through the bite of black flies of the genus *Simulium* that breed in fast-flowing water bodies [1]. It is estimated that nearly 37 million people are infected with onchocerciasis [2–4], and 200 million people are at risk of contracting the onchocerciasis infection [5]. Ninety-nine percent of the total global risk and burden is in Africa [6–10].

Onchocerciasis is a disease of major public health importance [11, 12]. The disease is the world’s second leading infectious cause of blindness after trachoma [13]. It is associated with reduced life expectancy [14, 15], and causes high mortality among onchocerciasis-blind people [16, 17] as well as epilepsy [14, 15, 18–20]. Onchocerciasis has also been associated with a variety of psychosocial and economic impacts. It results in social stigma of infected persons and their families [15, 21], disturbed sleep and reduced earnings among infected adults, poor school performance and a higher dropout rate among infected school-aged children [15, 22–27], and high health costs [4]. In fear of these occurring, communities migrate away from their fertile arable land [15, 28], cause drop in agricultural yields and perpetuate poverty.

Due to the high disease burden, the control of onchocerciasis has received considerable attention from various international organizations and donors. Concerted large-scale regional interventions carried out in the past four decades have been effective in bringing the disease burden under control; it is currently not a public health problem in most endemic areas in Africa [10, 29–31]. There is also established evidence that the disease is eliminated in some localized foci [28, 32–39]. Encouraged by these triumphs, the World Health Organization (WHO) established the target of onchocerciasis elimination in selected endemic countries by 2020 [40] and in remaining African countries by 2025 [41]. There is also international commitment to achieve the goal of disease elimination, as illustrated by the adoption of the World Health Assembly Resolution on NTDs (WHA 66.12) and endorsement of the London Declaration on NTDs of 2012 by pharmaceutical companies, donors, national governments, and non-governmental organizations (NGOs) [42]. In 2016, the WHO has published guidelines for stopping mass drug administration (MDA) and verifying the interruption of transmission and elimination of human onchocerciasis [43]. Onchocerciasis endemic countries have revised their plans, established a goal of disease elimination in line with an evidence based decision to stop MDA and verify elimination, incorporated it into their NTDs national master plans, and formed national onchocerciasis elimination committees to assist with and track elimination activities.

There are, however, many challenges to achieve the elimination of onchocerciasis in Africa [44–49]. This review examines the key challenges related to the elimination of onchocerciasis by 2020–2025 in Africa, and proposes recommendations to overcome the challenges and accelerate disease elimination. We believe that the review will help to inform many of the national programs working on the elimination of onchocerciasis in Africa.

## Main text

To find relevant articles published in peer-reviewed journals, a search of PubMed and Google Scholar databases was carried out. Titles of articles and abstracts were reviewed. If found relevant full length articles were retrieved and accessed. Publications were included if the abstract or full content of an article focused on accomplishments and challenges of previous onchocerciasis control programs, and current status, progress and challenges of disease elimination in onchocerciasis endemic countries of Africa. Additional articles were identified and retrieved by looking at the references list of the publications. The search included only articles published in English language. Moreover, websites of ministries of health of endemic countries and international health organizations (WHO, the United States Agency for International Development, The Carter Center, and Sightsavers) were assessed to obtain relevant data on national elimination programs and technical documents, and reports of consultative meetings, respectively. Abstracts of conference proceedings, chapters of books, and press releases were also included.

### Previous regional interventions for onchocerciasis control and elimination in Africa

Rigorous control interventions have been carried out to control and eliminate onchocerciasis in the past four decades in Africa. These include the Onchocerciasis Control Programme in West Africa (OCP) and the African Programme for Onchocerciasis Control (APOC).

The initial effort to control onchocerciasis was through the OCP, which ran from 1974 until 2002 in 11 West African countries that were most prevalent for the disease [29]. The OCP was initially a vector control program that was later complemented with MDA after ivermectin was registered for human use and donated for onchocerciasis control in 1987 [50]. At the OCP’s end, onchocerciasis had been eliminated as a public health problem in all participating countries except Sierra Leone where operations were interrupted by a decade-long civil war, and 25 million hectares of land had been reclaimed for agricultural use [51].

The APOC was launched at the end of 1995 to eliminate the public health problem of onchocerciasis in 19 African countries outside the OCP zone [2, 52, 53], which had more than 80% of the global burden of the disease [54, 55]. In 2007, the APOC received the mandate to assist any of the OCP countries as required. The primary activities of the APOC were to map out the endemicity of onchocerciasis and detect high transmission areas eligible for mass treatment [15]. For a sustained and cost-effective distribution of ivermectin, the APOC employed annual community-directed treatment with ivermectin (CDTI) as its strategy [15, 56–58]. It covered more than 190 000 communities, mainly in remote and hard-to-reach rural areas that were poorly serviced by health services, and lacked sufficient financial and human resources. This program effectively controlled the public health problem of onchocerciasis [2, 30, 31, 51, 59–63]. The rationales and historical milestones of these two regional program are summarized in Table 1.

**Table 1:** Summary of onchocerciasis control programmes in Africa: countries covered, objectives, strategies and achievements made at their closure

Program	Countries covered	Objective	Strategies	Achievements	References
OCP (1974–2002)	Benin, Burkina-Faso, Cote d’Ivoire, Ghana, Guinea, Guinea-Bissau, Mali, Niger, Senegal, Sierra Leone, Togo (SIZ in Togo and Sierra Leone for the period 2002–2007)	To eliminate onchocerciasis as a disease of public health importance and an obstacle to socio-economic development, and to ensure participating countries can maintain achievement	(1) Vector control, (2) Both vector control and CDTI, (3) CDTI only (in specific countries or areas)	Eliminated public health impact of onchocerciasis, Reclaimed 25 million km^2^ abandoned land for agriculture, Prevented 600 000 cases of blindness, Freed 18 million children from risk of blindness Infection disappeared from an estimated 1 million individuals Enhanced human resource capacity building via training of more than 400 professional staff Reduced disease associated social stigma 20% economic rate of return	[29–31, 50, 51, 64–71]
APOC (1995–2015)	Angola, Burundi, Cameroon, Central African Republic (CAR) , Chad, Democratic Republic of Congo (DRC), Equatorial Guinea, Ethiopia, Gabon, Kenya, Liberia, Malawi, Mozambique, Nigeria, Rwanda, Uganda, Tanzania, Sudan	To eliminate onchocerciasis as a disease of public health importance in the remaining endemic countries in Africa	The establishment of sustainable CDTI and vector control with environmentally-safe methods where appropriate.	Eliminated onchocerciasis as a disease of public health importance Saved 19 million disability-adjusted life years during its 20-year existence 20% decline in the disability-adjusted life year burden of onchocerciasis between 2005 and 2015 at a cost of only USD 27 per disability-adjusted life year Evidences of elimination of onchocerciasis in some localized foci	[2, 3, 15, 28, 33–39, 56, 57, 59–62, 69, 72]

*CDTI* Community-directed treatment with ivermectin, *OCP* Onchocerciasis Control Programme in West Africa, *USD* United States dollar

### Current status of onchocerciasis elimination in Africa

**Fig. 1:**
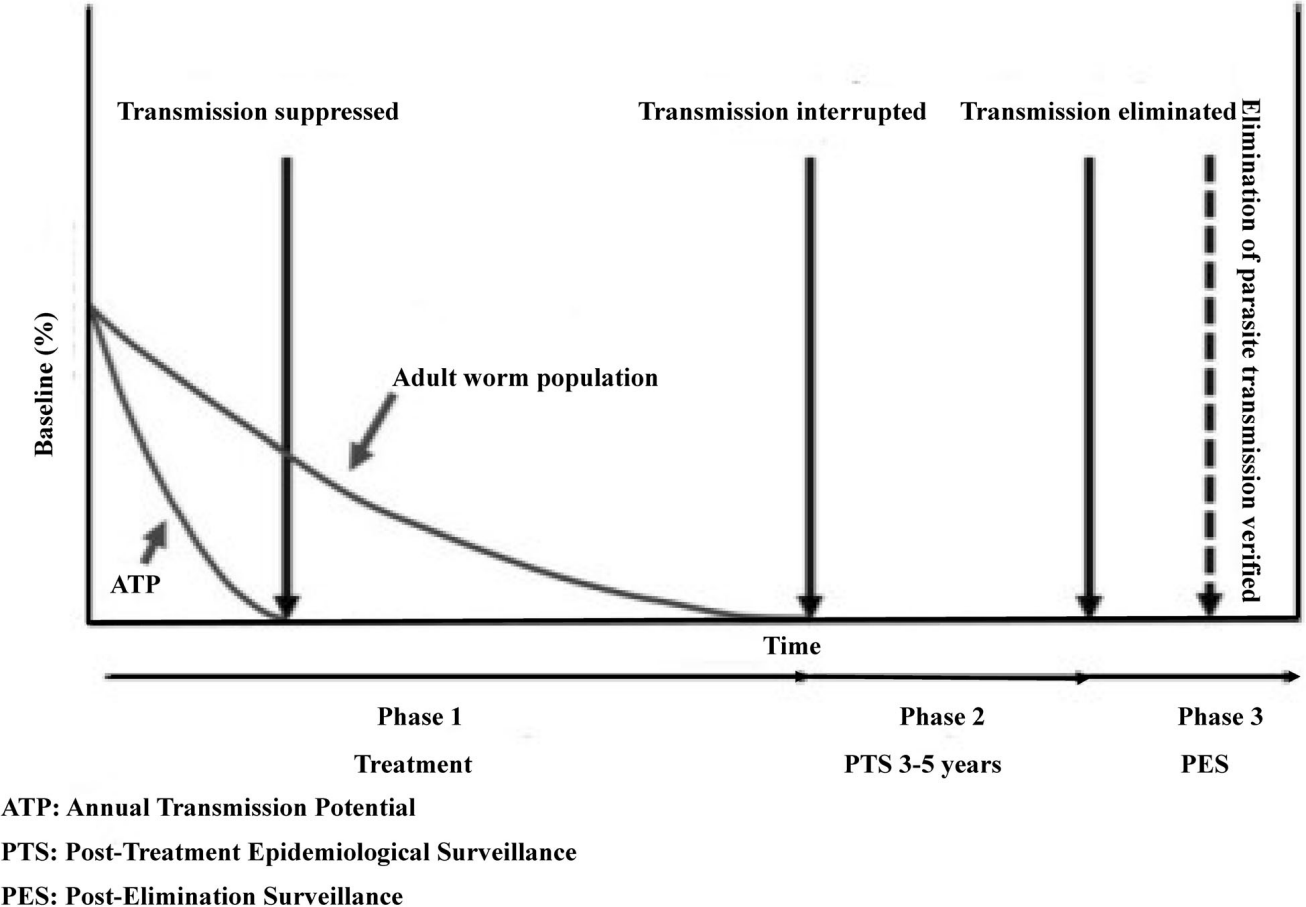
Phases in the elimination of human onchocerciasis (Source: [43])

After the APOC ended in 2015, it was replaced with the Expanded Special Project for Elimination of NTDs (ESPEN), which includes onchocerciasis elimination [63, 73, 74]. In accordance, onchocerciasis-endemic countries have shifted their policy from control to elimination and set an ultimate goal of interrupting parasite transmission from their territory. Elimination of onchocerciasis is defined as the “reduction of *O. volvulus* infection and transmission to the extent that interventions can be stopped, but post-intervention surveillance is still necessary” [75]. The WHO has published guidelines for stopping MDA and verifying the interruption of transmission and elimination of human onchocerciasis, which comprises three phases [43]:

- **Phase one** is a phase of active transmission and MDA intervention, characterized by regular ivermectin treatment with a minimum requirement of 80% therapeutic coverage of the eligible population for 12–15 years or longer in order to reach a point where transmission of the parasite can no longer be sustained. This has been supplemented with vector elimination efforts in selected onchocerciasis foci in Equatorial Guinea, Uganda, and Tanzania. At the end of this phase, programs perform epidemiological and entomological evaluations to demonstrate interruption of parasite transmission in order to stop MDA and move to the next phase. This includes testing children aged under 10 years and testing the heads of black flies for *O. volvulus* deoxyribonucleic acid (DNA) using Ov-16 enzyme-linked immunosorbent assay (ELISA) and O-150 polymerase chain reaction (PCR) (pool screening), respectively.
- **Phase two** is post-treatment surveillance (PTS) for new infections, lasting for 3–5 years. O-150 PCR testing is used to confirm the interruption of *O. volvulus* transmission in black flies. When O-150 PCR results are at or near the threshold (< one infected black fly in 2000 total flies), the Ov-16 serology test is used to confirm the interruption of parasite transmission in children. Moreover, Ov-16 serology is used to confirm the interruption of parasite transmission in some areas of Africa where vector elimination has been achieved. If confirmed the program enters the last phase.
- **Phase three** is a period of post-elimination surveillance to detect possible reintroductions of onchocerciasis [43]. This phase confirms permanent interruption or elimination of the disease in a defined geographic area [76]. The WHO’s three-phase elimination guidelines are briefly depicted in Fig. 1.

It is acknowledged that the initial goal of elimination of onchocerciasis as a disease of public health importance has been achieved in endemic areas of Africa including even where the endemicity of the disease was extremely high. The feasibility of elimination of onchocerciasis has also been confirmed in limited endemic foci in the last decade [28, 32–39].

### The major challenges relating to onchocerciasis elimination in Africa

In spite of this evidence that assures that the elimination of onchocerciasis from the continent is feasible, there are challenges regarding the accomplishment of this by 2025 [44–49]. The major challenges pertaining to the elimination of onchocerciasis in Africa are:

- incomplete elimination mapping of all transmission zones;
- co-endemicity of onchocerciasis and loiasis;
- possible emergence of ivermectin resistance;
- uncoordinated cross-border elimination efforts;
- conflict and civil war;
- suboptimal program implementation; and
- technical and financial challenges.

#### Incomplete elimination mapping of all transmission zones

In the control era, the primary task was to define the geographic distribution of onchocerciasis and delineate potentially endemic communities [77–80] via the rapid epidemiological mapping of onchocerciasis (REMO) strategy [81]. Areas with a prevalence of infection greater than 20% nodule prevalence in adult men in a community were defined as high-risk areas (mesoendemic and hyperendemic). These areas were associated with a significant risk of onchocerciasis disease [31] and it was decided that mass ivermectin treatment should be implemented forever [10, 79]. Indeed, successful progress is being made to interrupt transmission of the parasite in these areas [82]. The challenge lies with areas that are considered hypoendemic and nonendemic, where there is less than 20% onchocercal nodule prevalence [31, 83], which were excluded from treatment [84, 85] as it was decided that the level of infection did not constitute a serious public health problem. Now, the shift toward disease elimination has brought a new mapping challenge [86], as these disregarded hypoendemic areas might have contributed to sustained transmission and spread of infection [87–89], and become an impediment to disease elimination [90, 91]. Furthermore, despite a number of countries devising their own mapping strategy, a standardized common elimination mapping guideline is not yet available to complete the process.

#### Co-endemicity of onchocerciasis and loiasis

**Fig. 2:**
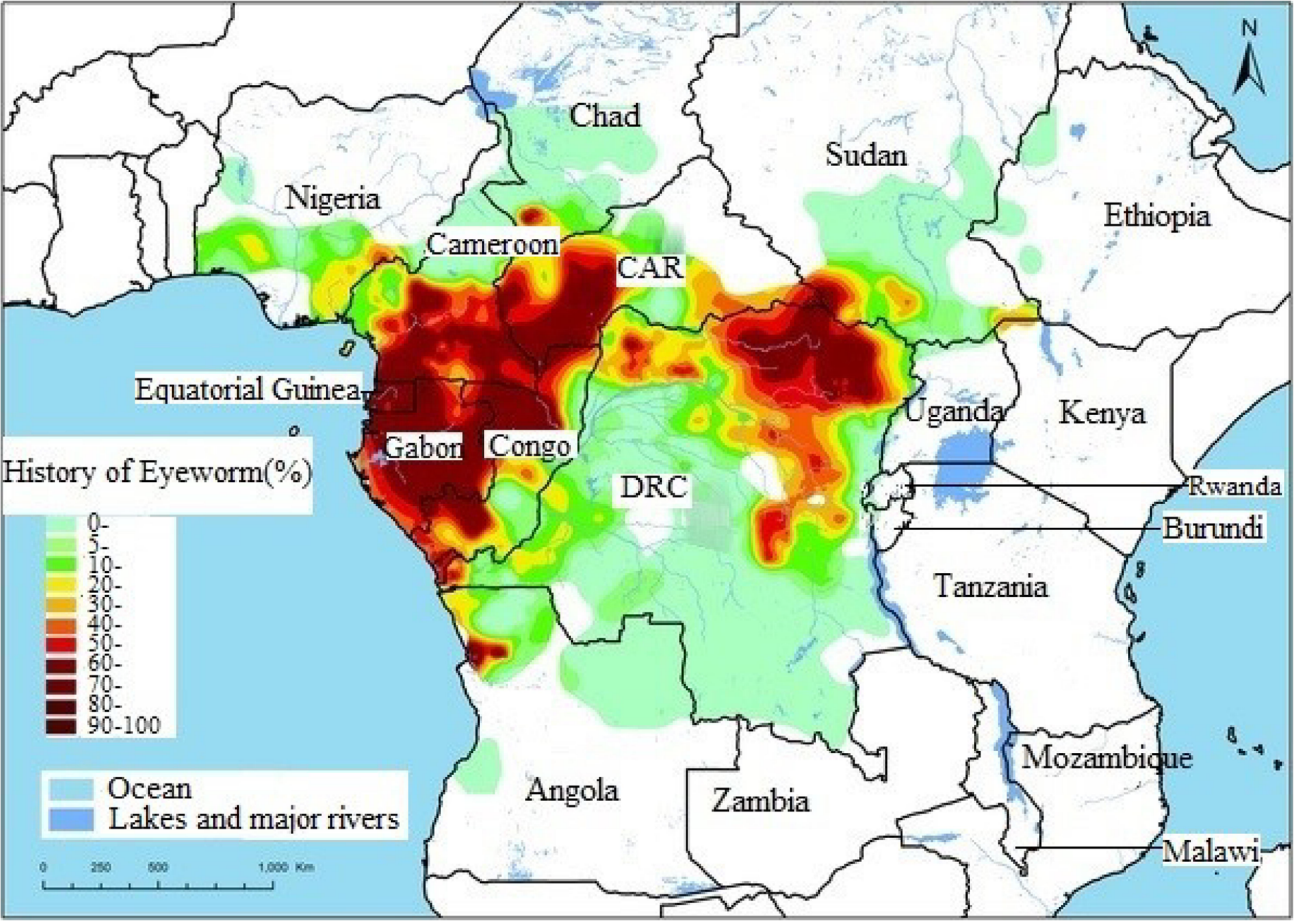
Map showing the estimated prevalence rates of *Loa loa* in Africa throughout history (Source: [92])

Loiasis is a filarial disease caused by the nematode parasite *Loa loa* [92]. It is estimated that 12–13 million people are infected in Africa [70]. The rapid mapping of parasite distribution revealed that there is a high prevalence of loiasis in two large foci in Africa, namely the west and east foci. The west focus includes southeast Nigeria, south Cameroon, Equatorial Guinea, Gabon, west Congo, the coastal plains of Angola, Bas-Congo in the Democratic Republic of the Congo (DRC), west Central African Republic (CAR), and south Chad. The east focus covers east CAR, south of South Sudan, and northeast DRC [92]. A small focus is found at the boundary between Kenya and South Sudan. The areas with low prevalence stretch from southwest Benin to west Ethiopia, and from north Angola to central Chad [44]. Figure 2 is map of estimated prevalence of *L. loa* in Africa throughout history.

*L. loa* causes ocular and systemic manifestations with minor effects on the quality of life [44]. The issue is that in areas co-endemic with onchocerciasis, ivermectin treatment of individuals harbouring high *L. loa* microfilariemia causes serious neurological adverse events that might lead to death [85, 90, 93–96]. The risk of severe adverse events is high when the microfilaria load exceed 8000 microfilaria/ml, and the reaction is more severe if the load exceeds 30 000 microfilaria/ml [93, 97–99]. As a result, millions of people living in areas where loiasis is co-endemic with onchocerciasis are left untreated with the standard strategy of community-wide ivermectin treatment [49, 85, 92]. Even in areas where treatment has been conducted under close surveillance to manage severe adverse effects, there is a high refusal rate to swallow the drug [74, 99] due to fear of risk of adverse events. This has contributed to the sustained transmission, reintroduction of the parasite into previously eliminated areas [49], and finally to it posing a major obstacle in the elimination of onchocerciasis [10, 85, 93, 94, 96, 100].

#### Possible emergence of ivermectin resistance

Ivermectin has long been used to control onchocerciasis [101, 102]. It is an effective microfilaricidal drug with a partial macrofilaricidal property when used repetitively: annually, semi-annually, or quarterly [103–105]. It should be noted that, despite several rounds of treatment, there are reports of *O. volvulus* responding poorly to the anti-fecundity effect of ivermectin in Ghana [106–109] and Cameroon [110, 111]. These observations raise the possibility that ivermectin resistance is emerging. This is supported by several parasite genetic studies that showed the occurrence of polymorphisms or changes in specific genes responsible for suboptimal responses in parasite populations exposed to several years of ivermectin treatment [112–120]. In 2017, a genome-wide analysis of *O. volvulus* revealed that evolution of suboptimal responses to ivermectin is determined by selective sweeps of pre-existing quantitative trait loci with many genes contributing in a polygenic manner [121]. Researchers examining human genes affecting drug response and clinical outcomes have reported that host genetic polymorphisms might be attributed to a varied response to ivermectin treatment in Ghana [122]. This phenomenon could jeopardize the goal of disease elimination, as ivermectin is still being used as the drug of choice in the elimination of onchocerciasis in Africa.

#### Uncoordinated cross-border elimination efforts

As onchocerciasis is extensively prevalent in a wide range of African countries, there are several cross-border issues that affect efforts of national programs working to achieve disease elimination [123]. These include international boundaries transversing disease transmission zones, and human population and vector migration [70, 89].

Endemic countries, mainly those with high-risk areas [1], have several shared transmission zones across their national borders [1, 70, 123]. This is because rivers, which offer breeding sites for black flies, often form borders between countries [30]. Elimination efforts along borders of countries with shared transmission zones might be uncoordinated [123] and MDA activities might be at different stages of implementation. This might therefore contribute to sustained transmission of infection and hinder disease elimination. For example, despite good progress made in achieving interruption of transmission in a number of foci in Uganda, recurrent transmission of onchocerciasis from cross-border areas of the DRC and South Sudan, where onchocerciasis is still endemic, poses a challenge [10, 91, 124, 125]. A similar problem of cross-border transmission of onchocerciasis has been reported in Malawi and Guinea-Bissau, with a possible source from adjacent border areas of Mozambique and Guinea, respectively [86]. In addition to international cross-border issues, internal administrative border issues within a country can also lead to significant challenges in the elimination of onchocerciasis [126] as CDTI projects in a country could be in different stage of programme implementation. Table 2 outlines the endemic countries in Africa and their respective cross-border endemic locations.

**Table 2:** Onchocerciasis endemic countries and their shared cross-border locations (Source: [126])

Country	Shared cross-border endemic location with
Angola	DRC
Benin	Togo, Nigeria, Burkina Faso
Burkina Faso	Mali, Cote d’Ivoire, Ghana, Togo, Benin, Niger
Cameroon	Nigeria, Chad, Congo-Brazzaville, CAR
CAR	Cameroon, Chad, South Sudan
Chad	Cameroon, CAR, South Sudan
Congo-Brazzaville	Cameroon, Gabon, DRC
Cote d’Ivoire	Liberia, Burkina Faso, Ghana, Mali
DRC	Uganda, Angola, CAR, Congo, South Sudan, Zambia
Equatorial Guinea	Cameroon, Gabon
Ethiopia	Sudan, South Sudan
Gabon	Cameroon, Congo
Ghana	Cote d’Ivoire, Burkina Faso, Togo
Guinea	Guinea Bissau, Senegal, Mali, Sierra Leone, Liberia
Guinea Bissau	Senegal, Guinea
Kenya	South Sudan , Tanzania
Liberia	Sierra Leone, Guinea, Cote d’Ivoire
Malawi	Mozambique
Mali	Senegal, Guinea, Cote d’Ivoire, Burkina Faso, Benin, Niger
Mozambique	Malawi, Tanzania
Nigeria	Benin, Cameroon, Chad, Niger
Niger	Burkina Faso, Benin, Nigeria, Chad, Mali
Senegal	Guinea-Bissau, Guinea
Sierra Leone	Guinea, Liberia
South Sudan	Uganda, Ethiopia, Sudan
Sudan	Ethiopia, South Sudan
Togo	Benin, Ghana, Burkina Faso
Tanzania	Mozambique, Burundi, Kenya
Uganda	DRC, South Sudan

*CAR* Central Africa Republic, *DRC* Democratic Republic of the Congo

Human population and black fly migration across national borders also has a significant impact on the elimination of onchocerciasis in Africa [70, 127]. There is seasonal population migration from one country to another, or from one area of a country to another, for several activities such as fishing, farming and mining. Infected migrating people either spread the infection into disease-free areas or disease-free migrating persons acquire infections from an onchocerciasis-endemic area to which they arrive. For instance, reinfection of a community nearly free from onchocerciasis in Burkina Faso was due to migration of infected persons from neighbouring onchocerciasis-endemic zones of Côte d’Ivoire [128].

In addition to human migration, cross-border vector flying contributes to the dispersal of parasites and reinvasion of disease-free areas, as observed in the previous OCP areas [75, 127, 129, 130]. Savanna black flies can travel up to 600 kilometers [131] with a wind assisted flight and maintain transmission at the point of their arrival despite a presence of local intervention [127]. A group of researchers indicated that migrating flies were one of the possible factors for the reintroduction of infection into the previously disease-free OCP areas of Burkina Faso [132]. Similarly, cross-border migration of vectors might have partly contributed to the persistence of infection and ongoing transmission of *O. volvulus* in the river basin areas of northern and central Togo despite decades of vector control and MDA intervention [133]. Apart from international borders, human and black fly migration can also carry and disperse parasites between local foci within a country [74].

#### Conflict and civil war

The presence of conflict and civil war in some African countries is another impediment in the elimination of onchocerciasis [9, 71]. Conflict interrupts interventions and weakens political support [9], and is thus responsible for the occurrence of new cases of onchocerciasis-related blindness every year [10]. Conflict and post-conflict African countries that have anticipated delayed achievement of disease elimination include South Sudan, the DRC, CAR, Angola, and Côte d’Ivoire [9, 70, 134]. In countries with such a crisis, the healthcare system is fragmented [135], and it is difficult to obtain access to deliver regular MDA and carry out entomological and epidemiological surveillances activities [136] for monitoring and evaluation of program intervention. For instance, in 2016, MDA was seriously disrupted and the number of treatments declined to 196 000 (from 462 000 in 2015) due to deteriorating security in South Sudan [137]. People are displaced from their original residences, marginalized from receiving ivermectin treatment on a regular basis [68], and may serve as reservoirs of infection [136].

#### Suboptimal program implementation

There are different programmatic factors that affect the elimination of onchocerciasis in Africa, including the extent of drug coverage (i.e. geographic and therapeutic), compliance to CDTI [138, 139], and frequency of mass ivermectin treatment [131]. Geographical coverage is defined as the proportion of administrative units treated out of the total number of units requiring treatment in a particular MDA program [140, 141]. It is an indicator of the scaling up of the MDA program in a country [140]. Therapeutic coverage is defined as the percentage of the eligible population that participates in a CDTI program in a given area [62].

**Fig. 3:**
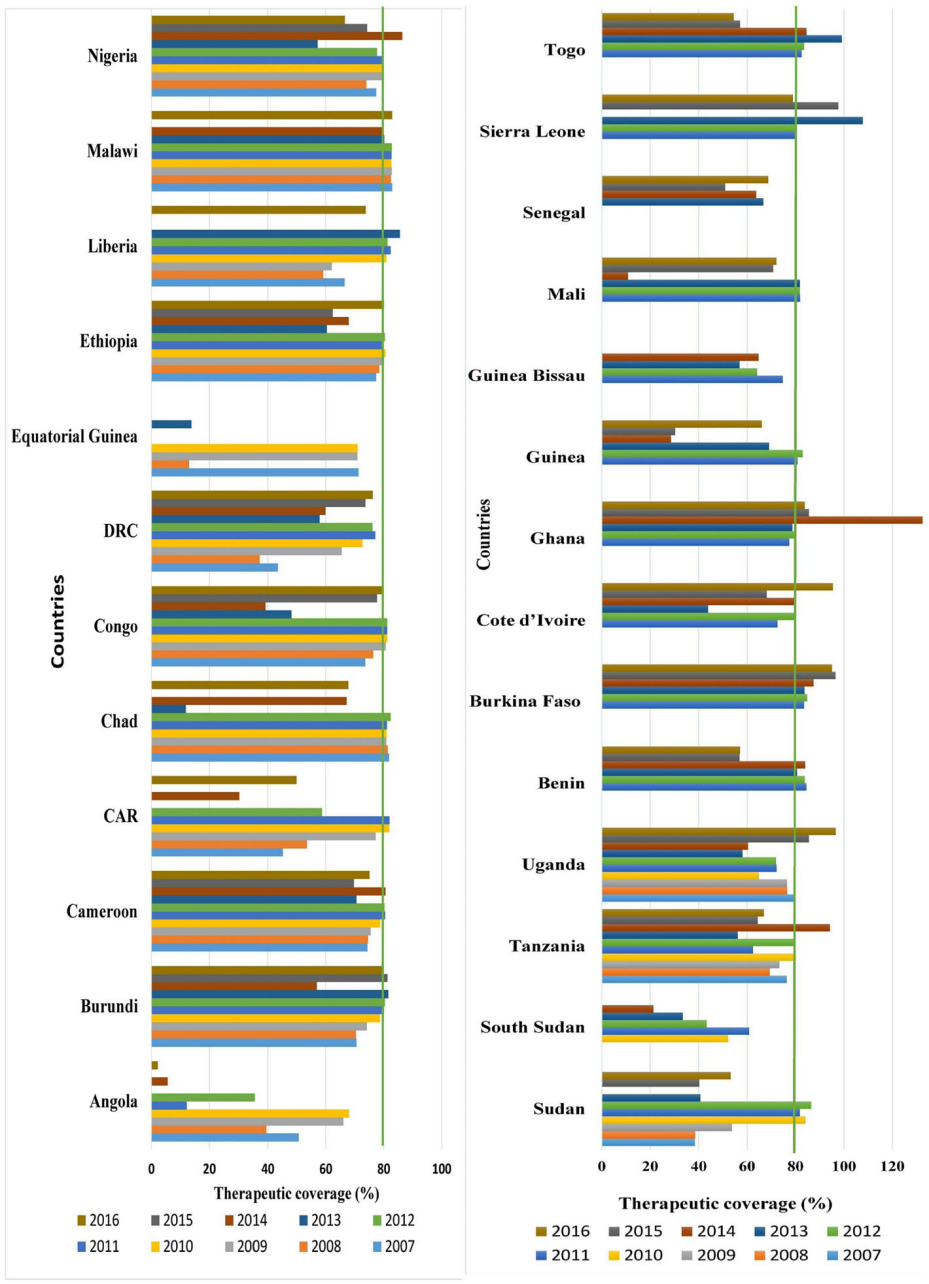
Temporal trends of therapeutic coverage of MDA in 26 onchocerciasis-endemic countries in Africa, 2007–2016 (Source: [143–152])

Achieving and maintaining high geographic and therapeutic coverage during each MDA round will help to shorten the period needed to interrupt parasite transmission [142]. Trends in ivermectin therapeutic coverage in 26 countries of Africa are presented in Table 3 and Fig. 3. According to the WHO’s Weekly Epidemiological Record, a total of 53 865 599 people received treatment in 2007 in Africa [143]. This number has increased each year, and a reported 132 502 932 people received treatment at least once in 2016 [143–152]. Therapeutic coverage was 65.4% in 2007, 61.2% in 2008, 73.1% in 2009, 75.8% in 2010, 77.4% in 2011, 76.4% in 2012, 59.6% in 2013, 65.3% in 2014, 60.5% in 2015, and 67% in 2016 (see Table 3 and Fig. 3). This shows that, continentally, the minimum threshold required for interruption of parasite transmission has not yet been reached. Though it is fluctuating, individual country data show that most countries have achieved the effective threshold required to control the disease over 10 years. Therapeutic coverage above the minimum disease elimination threshold (80%) was also reported: Malawi has achieved this in 8 years; Burundi and Burkina Faso in six; Chad in five; Congo, Ethiopia, Liberia, Benin, Sierra Leone, and Togo in four; and Cameroon, Ghana, Mali, Nigeria, Uganda, and Sudan in 3 years over 10 years. Angola, the DRC, CAR, Equatorial Guinea, and South Sudan had poor therapeutic coverage during the MDA period (see Table 3 and Fig. 3). Key factors for poor drug coverage that hamper reaching the recommended threshold include: co-endemicity with loiasis [135], shortage of drug supply [146], and logistical challenge of delivering ivermectin to endemic areas in a timely manner [10, 151], mainly to those living in remote and hard-to-reach endemic areas [44, 74, 153]. To achieve elimination as quickly as possible, adequate support needs to be provided to national programs with poor drug coverage to scale up their mass ivermectin treatment.

**Table 3:** Therapeutic coverage of MDA programme in 26 onchocerciasis endemic countries in Africa, 2007–2016 (Source: [143–152])

Country	2007	2008	2009	2010	2011	2012	2013	2014	2015	2016	Therapeutic coverage in 10 years			
											≥ 80	71.9–79.3	65.3–71.5	< 64.2
Angola	50.7 (414 965)	39.5 (282 913)	66.1 (423 391)	68 (711 276)	12.2 (131 487)	35.5 (430 329)	NA	5.6 (145 643)	0 (0)	2.2 (123 759)	-	-	2^a^	7^a^
Burundi	70.6 (860 416)	70.4 (899 564)	74.2 (1 044 371)	178.7 (1 146 033)	80.1 (1 176 498)	80.4 (120 443)	81.6 (1 245 115)	56.9 (1 286 570)	81.3 (1 352 797)	80 (1 358 226)	5^a^	2^a^	2^a^	1^a^
Cameroon	74.4 (4 427 481)	74.6 (4 647 473)	75.5 (4 809 180)	78.8 (5 300 025)	80.5 (5 403 559)	80.3 (5 500 491)	70.6 (6 176 064)	80.6 (7 203 643)	69.7 (7 572 216)	75.1 (8 161 467)	3^a^	5^a^	2^a^	-
CAR	45.2 (724 791)	53.5 (870 516)	77.2 (1 088 053)	81.9 (1 264 508)	82 (1 502 260)	58.7 (985 325)	0 (0)	30.3 (651 857)	NA	49.9 (1 240 157)	2^a^	1^a^	-	6^a^
Chad	81.8 (1 389 921)	81.4 (1 421 448)	80.9 (1 513 713)	81 (1 542 377)	81.1 (1 621 004)	82.4 (1 718 974)	11.9 (300 245)	67.2 (1 732 615)	NA	67.8 (2 588 316)	6^a^	-	2^a^	1^a^
Congo	73.6 (449 171)	76.4 (478 692)	80.7 (617 167)	81.2 (651 922)	81.2 (686 127)	81.2 (1 219 178)	48.2 (688 131)	39.2 (571 752)	77.7 (402 635)	79.3 (473 321)	4^a^	4^a^	-	2^a^
DRC	43.5 (9 230 951)	37.2 (9 534 666)	65.5 (17 704 257)	72.7 (20 290 244)	77.1 (22 403 957)	76.1 (23 126 855)	57.9 (24 536 180)	59.9 (26 049 139)	73.7 (29 751 168)	76.2 (31 374 142)	-	5^a^	1^a^	4^a^
Equatorial Guinea	71.3 (50 064)	13 (9294)	70.9 (56 902)	71 (57 735)	0 (0)	0 (0)	13.8 (11 840)	0 (0)	NA	NA	-	-	3^a^	5^a^
Ethiopia	77.4 (4 135 538)	78.5 (4 315 374)	80.1 (4 613 362)	80.6 (4 809 869)	79.3 (4 741 282)	80.4 (6 446 552)	60.4 (7 165 807)	67.9 (8 222 238)	62.4 (10 643 027)	80.1 (13 934 852)	4^a^	3^a^	1^a^	2^a^
Liberia	66.5 (2 442 161)	59.1 (3 302 966)	62.1 (1 288 496)	80.9 (2 003 343)	82.4 (2 419 509)	81.3 (2 388 812)	85.6 (2 646 567)	0 (0)	NA	73.8 (2 148 543)	4^a^	1^a^	1^a^	3^a^
Malawi	82.9 (1 546 433)	82.5 (1597659)	82.8 (1 638 355)	82.6 (1 666 048)	82.7 (1 718 966)	82.8 (1 758 924)	80.2 (1 777 145)	79.3 (1 813 432)	NA	82.9 (1 894 776)	8^a^	1^a^	-	-
Nigeria	77.4 (22 839 983)	74 (23 599 225)	80.1 (26 666 032)	80 (29116332)	79.4 (30 439 546)	77.7 (29 032 404)	57.2 (28 661 160)	86.4 (44 415 242)	74.3 (35 271 150)	66.6 (35 362 158)	3^a^	5^a^	1^a^	1^a^
Sudan	38.4 (1 499 137)	38.5 (1 999 030)	53.7 (3 011 429)	84.1 (329 702)	81.7 (329 702)	86.5 (146 468)	40.7 (177 015)	NA	40.2 (153 134)	53.2 (266 532)	3^a^	-	-	6^a^
South Sudan	NA	NA	NA	52.2 (2 981 506)	60.8 (3 467 340)	43.3 (2 473 693)	33.4 (2 271 979)	21.3 (1 485 042)	NA	NA	-	-	-	5^a^
Tanzania	76.3 (1 684 661)	69.3 (1 554 102)	73.3 (1 616 757)	80.1 (1 901 542)	62.4 (1 456 302)	79.2 (1 872 181)	56.1 (1 928 730)	94.2 (3 338 320)	64.4 (3 640 830)	66.9 (4 117 571)	2^a^	3^a^	3^a^	2^a^
Uganda	80 (2 169 926)	76.6 (2 203 148)	76.4 (2 328 352)	64.8 (2 031 079)	72.2 (2 749 364)	71.9 (2 359 914)	58.1 (2 504 625)	60.4 (2 687 991)	85.5 (1 921 562)	96.6 (1 908 449)	3^a^	4^a^	1^a^	2^a^
Benin	NA	NA	NA	NA	84.5 (2 745 061)	83.8 (2 768 062)	80.7 (2 764 755)	83.9 (2 952 152)	56.9 (3 651 804)	57.1 (3 795 689)	4^a^	-	-	2^a^
Burkina Faso	NA	NA	NA	NA	83.6 (176 936)	84.8 (186 040)	83.7 (187 732)	87.4 (202 009)	96.5 (244 178)	95 (242 786)	6^a^	-	-	-
Cote d’Ivoire	NA	NA	NA	NA	72.6 (1 333 662)	79.3 (1 219 178)	43.8 (1 001 818)	79.9 (1 865 273)	68.1 (2 107 463)	95.4 (2 929 230)	2^a^	2^a^	1^a^	1^a^
Ghana	NA	NA	NA	NA	77.4 (1 776 626)	79.7 (3 466 716)	78.6 (3 495 861)	132.5 (3 372 058)	85.5 (3 416 583)	83.8 (4 331 666)	3^a^	3^a^	-	-
Guinea	NA	NA	NA	NA	80.8 (2 517 391)	83 (223 6136)	69 (2 261 919)	28.5 (956 726)	30.3 (2 544 455)	66.1 (4 485 382)	2^a^	-	2^a^	2^a^
Guinea Bissau	NA	NA	NA	NA	74.7 (135 014)	64.1 (107 835)	56.9 (107 278)	64.7 (124 517)	0 (0)	NA	-	-	2^a^	3^a^
Mali	NA	NA	NA	NA	81.9 (3 925 970)	81.7 (3 956 909)	81.7 (4 149 706)	10.8 (566 480)	70.8 (3 609 017)	72.1 (3 988 774)	3^a^	1^a^	1^a^	1^a^
Senegal	NA	NA	NA	NA	NA	NA	66.7 (120 438)	63.8 (118 224)	50.9 (446 184)	68.7 (628 813)	-	-	2^a^	2^a^
Sierra Leone	NA	NA	NA	NA	80.3 (2 446 658)	80.2 (2 642 036)	107.7 (3 419 081)	0 (0)	97.7 (3 295 899)	78.8 (4 202 381)	4^a^	1^a^	-	1^a^
Togo	NA	NA	NA	NA	82.6 (247 7365)	83.6 (2 599 544)	99.1 (3 094 350)	84.5 (2 694 268)	57.1 (2 690 686)	54.5 (2 945 942)	4^a^	-	-	2^a^
Africa	65.4 (53 865 599)	61.2 (56 716 070)	73.1 (68 419 817)	75.8 (75 803 541)	77.4 (9 778 158)	76.4 (99 316 949)	59.6 (100 693 541)	65.3 (112 455 191)	60.5 (112714788)	67 (132502932)	-	4^a^	3^a^	3^a^

*CAR* Central Africa Republic, *DRC* Democratic Republic of the Congo, *NA* Data not reported

^a^Therapeutic coverage achieved in 10 years

Compliance is another critical programmatic issue that should be considered in the elimination of onchocerciasis in Africa. It refers to the frequency with which individuals comply to swallow the provided drugs [141]. It has been found that not all eligible members of a community swallow the drug they receive during a MDA campaign and a wide range of factors are affecting compliance to CDTI [135, 141, 154–169]. For instance, compliance to CDTI was associated with older age [165, 167], perceived individual’s risk of onchocerciasis infection [157, 162, 163, 165], perceived belief of benefits of ivermectin [154, 157, 159, 162, 167, 169], positive beliefs as community drug distributors (CDDs) are doing their job well [157, 163, 165], whereas being female and individual’s past experience of drug adverse effects were associated with non-compliance to CDTI [168]. There are also individuals in a community who fail to comply over several years of annual CDTI, known as systematic non-compliers. They account for more than 10% of a population [166] that might act as a reservoir of infection, which maintains transmission of the parasite [141, 166, 168], thus making interrupting the transmission challenging [74].

Frequency of mass ivermectin treatment is another programmatic factor that affects the elimination of onchocerciasis. When control was the agenda of control programs, annual treatments appeared to be sufficient to eliminate the public health and socioeconomic impact of onchocerciasis. With the shift to the agenda of elimination, the annual mass ivermectin treatment approach is questionable [131], particularly in settings with high pre-control endemicity and areas where CDTI has recently started [9, 49]. For instance, a large-scale epidemiological evaluation of CDTI areas showed that more than 20 years of annual CDTI with adequate coverage was not able to interrupt parasite transmission in Touboro, north Cameroon, an area with the highest recorded pre-control endemicity level [30]. Model-based comparisons of annual and biannual treatments also concluded that biannual treatments would reduce the number of treatment years by one third compared to annual treatments in highly endemic areas [49, 142, 170]. Biannual and/or quarterly MDA was found to be critical for the interruption of parasite transmission in Latin American endemic countries [30].

#### Technical and financial challenges

With the conclusion of the APOC and shift to ESPEN in 2016, interventions for other NTDs are integrated into the program for onchocerciasis elimination [73]. This has brought changes to the well-functioning community MDA approach for onchocerciasis and added a load to the minimally educated and unpaid CDDs [171]. There are also technical challenges to integrating disease-specific MDA programs [172] with different implementation units into a combined NTDs platform [45, 173, 174]. Moreover, thorough scientific evidence is needed to confirm interruption of transmission and verification of elimination using specialized and quality-assured laboratory facilities and diagnostic tools [71]. Well-trained field entomologists are also required to collect entomological data to monitor and evaluate program performance.

Adequate funding is always crucial to any effort toward the control or elimination of any disease. In the past four decades of onchocerciasis control, endemic countries relied on external financial aid from international donor organizations. The core principle of the APOC was to create government-supported self-sustainable community programs. However, it is evidenced that governments of endemic countries were often unable to offer sufficient financial resources to their onchocerciasis projects in the APOC era [56, 175, 176]. They even failed to do so when the APOC came to its end in 2015 [173]. This was complemented by decreased donors’ interest to support the program [177]. The current program, ESPEN, is also not a major funding organization. It was established to ensure that endemic countries take ownership of their national programs [178] and to coordinate the technical support for the interventions of five NTDs including onchocerciasis [30, 73]. Inadequate funding is, therefore, a challenge that could prevent the realization of complete elimination of onchocerciasis in Africa.

Sustainability of CDTI necessitates continued education and mobilization of the community, supervision, communication, and human resource capacity-building via refresher or regular training of community leaders, CDDs, frontline health workers, district managers, regional-level implementers, and national program managers during every round of MDA [45]. These are continuing costs to the elimination programs [45] that need adequate financial resources as a proportion of health budgets [141], particularly in areas that commenced to implement biannual mass treatments to achieve their elimination target [9, 179]. Another critical issue is maintaining the motivation and willingness of unpaid and volunteer community workers who distribute the donated drug in their communities. Low motivation and high attrition rates of CDDs have been identified in many endemic countries [135, 180]. Lack of incentive or remuneration has been identified as one of the causes [135]. The work of CDDs is even more overloaded in the current ESPEN with integrated interventions for NTDs [181, 182], which might eventually diminish their performance [171]. Given the value of their role in the elimination of onchocerciasis, incentives have to be provided to keep CDDs motivated to perform their job effectively [183]. It has also been found that incentives in various forms for community volunteers have been shown to enhance program delivery and community participation [181, 184, 185].

Moreover, as country programs advance toward the endpoint, it is essential to implement complete elimination mapping in previously untreated or hypoendemic areas (which are geographically vast and hard to reach for health services) [186] and follow the progress of elimination efforts [187]. These two key activities demand the utilization of the WHO’s recommended tools and costly laboratory operations [179]. For example, in the course of eliminating onchocerciasis in Uganda, the annual cost of laboratory procedures was about United States dollars (USD) 35 000–40 000 [177]. This might be higher in countries that do not have their own national molecular laboratory settings for processing samples. Therefore, given the increased scope of elimination activities, financial constraints remain among the chief concerns of national programs for the elimination of onchocerciasis in Africa [96, 173].

### Recommendations to overcome the challenges and accelerate onchocerciasis elimination

The elimination of onchocerciasis by 2025 requires the criteria for stopping MDA to be achieved by latest 2022 [75] so that PTS can begin [188]. Countries need to evaluate and regularly monitor their national programs and identify the major challenges impeding the targeted interruption of parasite transmission. Recommendations to overcome the aforementioned challenges and suggested actions to accelerate the elimination of onchocerciasis by 2025 include:

- a need for complete disease elimination mapping;
- a need for collaborative elimination activities between national programs;
- a need for a different drug distribution approach in conflict-affected areas;
- a need for routine monitoring and evaluation of MDA programs;
- a need for implementing alternative treatment strategies (ATSs) in areas with elimination anticipated beyond 2025; and
- a need for strong partnerships and continued funding.

#### A need for complete disease elimination mapping

The transition from the goal of control to elimination of onchocerciasis from all endemic areas of Africa [85] necessitates the revision of deployed approaches in order to define the distribution of onchocerciasis [179, 189] and thus the implementation of MDA [82]. The decision to implement mass treatment needs to be based on the presence or absence of the transmission of infection [88]. All geographic areas where sustained local transmission of the parasite is likely but that were previously excluded from control programs as nonendemic or hypoendemic need to be mapped [127, 189]. The WHO recommends the Ov-16 serological test [82, 88, 127] for onchocerciasis elimination mapping to determine eligibility for MDA, as this would help to detect a status of infection and parasite transmission at a low level of endemicity [86, 127]. If it is proven that there is sustained transmission of the parasite [189], programs should implement an appropriate intervention [178, 189]. The onchocerciasis elimination mapping, therefore, helps to focus intervention in areas where parasite transmission is definitely happening [89]. According to the WHO Onchocerciasis Technical Advisory Subgroup 1st meeting report of 2018, the provisional threshold for commencing mass ivermectin treatment is set at 2% Ov-16 seropositivity [82]. The Ov-16 serology for elimination mapping offers a more sensitive indicator of infection [190]. However, there are concerns pertaining to the tool measuring infection and transmission as it does not differentiate active infection from previous exposure to infection with full certainty. It is not clear how to interpret serological data from surveys in hypoendemic areas. There are also debates among experts on the criteria for deciding and implementing MDA. It is, therefore, necessary to establish a measurable threshold to identify the status of local transmission in hypoendemic areas by considering the tool’s test performance [127, 189]. The WHO needs to avail a standardized onchocerciasis elimination mapping strategy that guides national elimination programs in Africa to accomplish their disease mapping.

#### A need for collaborative elimination activities between national programs

Cross-border foci will require a high degree of political, managerial, and scientific coordination between the respective parties involved to ensure complete success in eliminating onchocerciasis [44]. National elimination programs of neighbouring countries need to undertake coordinated and collaborated cross-border elimination activities [71, 92] to ensure that cross-border issues do not affect the progress toward elimination. Key activities that could be carried out in a collaborative manner between national programs include: routine and continuous communication for common understanding [126], reaching all their adjoining endemic areas of countries with MDA, harmonizing MDA and surveillance activities, and sharing of data, best practices, and successful approaches [71, 123]. For example, cross-border collaboration in the control of onchocerciasis in Mano River Basin countries is exemplary in that it provides valuable lessons to carry out an effective MDA for the elimination onchocerciasis and other NTDs [123] which can be applied for other African countries faced with cross-border transmission of infection. Moreover, routine entomological studies need to be carried out to evaluate the risk of reintroduction of infection via vector migration in areas where cessation of ivermectin distribution is being considered [75].

#### A need for a different drug distribution approach in conflict-affected areas

There is disruption of the population structure due to extensive migration and resettlement in conflict-affected areas [141]. Thus, the MDA strategy carried out in conflict areas needs to be different from the CDTI approach in usual situations [74]. MDA in a conflict area could be carried out through collaboration with local NGOs or humanitarian organizations, whose volunteers are often present in war zones to achieve an improved geographic coverage of ivermectin [74]. Moreover, migrant and displaced people need to be outreached during MDA campaigns at their destinations via appropriate strategies [139]. For instance, either CDDs from hosting areas should be trained to include migrant and displaced people during the drug distribution period or drug distributors from among migrant/displaced people need to be trained.

#### A need for routine monitoring and evaluation of MDA programs

To interrupt the transmission of onchocerciasis, monitoring and evaluation of MDA impact on infection and transmission, and evaluation of the decline in infection levels is crucial [88]. This will help to identify areas that are not performing well in an elimination program [88]. The WHO has published guidelines for stopping MDA and the evidence required for verification of interruption of transmission in 2016. The guidelines specify that routine entomological and serological monitoring and evaluation should occur at least every 4–5 years according to local regional guidelines [43]. Despite this, there are issues with the criteria for evaluating progress toward elimination, which need revision. To determine whether MDA can be safely stopped and detect a prevalence of less than 0.1% threshold, the 2016 WHO guidelines state that a sample size 1000–2000 children aged below 10 years need to be tested [43]. However, the Ov-16 ELISA has a sensitivity of 80% and specificity of 97% [192]. A test with 97% specificity cannot measure 0.1%. It is not therefore possible to reach this threshold with the test performance (specificity) of the current diagnostic tool [191]. To be successful at detecting less than 0.1% threshold needs a test that achieves greater than 99.9% specificity [191], and a large sample size is needed [74]. Hence, with this threshold, areas where transmission has been interrupted will fail to meet the criterion and demand to carry on unwarranted MDA [192]. As many national onchocerciasis programs with several years of CDTI under their belt are now preparing to apply this guidance, it is important that the current threshold for MDA cessation be revised [191].

Furthermore, successful elimination of onchocerciasis requires routine monitoring of factors that may affect program sustainability and evaluation of progress of CDTI interventions. Drug coverage verification surveys need to be carried out carefully and on a regular basis following campaigns [140, 194, 195] to identify communities with low therapeutic coverage [193, 196], and then to enable changes to be made to MDA implementation [140]. The participation of the community and compliance to CDTI need to be monitored, and factors associated with non-compliance should be identified. Emphasis should be given to identifying systematic non-compilers. A community’s awareness and correct perception of onchocerciasis and MDA is important for successful implementation and sustainability of a CDTI program [197]. Community-based social research is also needed to determine the community’s awareness and perception of onchocerciasis [74] and other factors contributing to program weakness [45]. Community awareness should be sustained through consistent social mobilization and health education [141]. Moreover, it is worth bearing in mind that long-term use of ivermectin could induce selection pressure on the parasite genome for the occurrence of drug resistance. This should be monitored regularly by carrying out genetic-based studies using molecular markers. Above all, it should be emphasized that national elimination programs need to expand drug coverage to all geographic areas and eligible members of communities to achieve interruption of parasite transmission and ultimately ensure disease elimination [151, 152].

#### A need for implementing ATSs in areas with elimination anticipated beyond 2025

Endemic areas where it is anticipated that interruption of transmission will occur after 2025 may require ATSs to accelerate infection decline toward elimination [127]. These include enhanced CDTI, use of other drugs, complementary vector control, and test-and-treat strategies [46, 85, 139].

An enhanced CDTI (biannual or pluriannual) approach could be used to target complicated areas, for instance, those where annual CDTI has not interrupted transmission after a long time of treatment [127], and those with no or only a short history of treatment [49]. A similar approach could also be followed in communities with poor ivermectin responses and areas where elimination activities are frequently interrupted due to conflict and civil unrest [139].

Alternative drugs or therapies can also be applied to areas where CDTI cannot be implemented effectively [71, 85, 127], or in areas where program implementation is considered to be insufficient [139]. New therapies to eliminate onchocerciasis have also been developed [46, 139]. After decades of clinical trials [198–200], the United States Food and Drug Administration (US FDA) has approved moxidectin for the treatment of onchocerciasis [201–204]. Moxidectin has superior clinical efficacy [183, 198] and a better safety profile compared to ivermectin [46, 200]. This could potentially improve a community’s participation during MDA, particularly regarding systematic non-compliers [205]. If made available to endemic countries, moxidectin could help to accelerate the progress toward onchocerciasis elimination [204], including in those areas with suboptimal responses to ivermectin [200]. Another alternative antibiotic targeting *Wolbachia* endosymbionts of *O. volvulus* could also be used within test-and-not-treat strategies [46], primarily in areas co-endemic with loiasis [206]. This can be achieved using doxycycline, which is currently the only usable macrofilaricide [207]. Vector control, using WHO approved and environmentally safe insecticides [188], is also a known alternative strategy for eliminating the black fly or reducing its density to levels where the disease is eliminated [208]. Therefore, localized vector control could be considered in high-transmission settings where MDA alone is not sufficient to interrupt transmission of the parasite [49] and areas where onchocerciasis is co-endemic with loiasis [85]. The WHO/APOC guide for decision making and implementation of vector control as ATSs for elimination of onchocerciasis (WHO/MG/15.22) also recommends the implementation of vector control as an alternative strategy to accelerate the elimination of onchocerciasis [188].

#### A need for strong partnerships and continued funding

The successes of previous control programs can be, at least partly, attributed to strong partnerships among different stakeholders, including: international donors; the WHO, with its technical guidance and support; Merck pharmaceutical, with its donation of Mectizan® and all importation costs covered; governments of endemic countries; NGOs working closely with governments of endemic countries, in terms of their financial and technical support; and communities. All these bodies and groups contributed to the elimination of onchocerciasis as a public health problem in endemic areas of Africa [178].

The relevance of strong partnerships and a range of donors remains vital [209]. The current ESPEN needs to work collaboratively with major international funders as well as with other stakeholders to maintain the achievements of previous programs, and to offer both technical and financial support to eliminate onchocerciasis [74]. Governments of endemic countries and their NGO partners need to take full ownership of their national elimination programs by allocating and mobilizing adequate resources for the intended goal. A strong commitment is compulsory from volunteer CDDs, frontline health workers, and beneficiary communities to reach the last mile.

## Conclusions

Great strides have been made over the past decades to control and eliminate onchocerciasis in Africa through devoted regional programs, which have been largely successful in most endemic countries. Elimination of onchocerciasis as a disease has also been achieved in limited localities in Africa, and this has provided optimism for a complete elimination of the disease from the rest of Africa by 2025. However, there are numerous challenges that hinder the existing efforts to eliminate onchocerciasis from Africa. The major challenges include an incomplete mapping of all transmission zones, co-endemicity of onchocerciasis and loiasis, possible emergence of ivermectin resistance, uncoordinated cross-border elimination efforts, conflict and civil unrest, suboptimal program implementation, and technical and financial challenges.

It is, therefore, recommended that the impact of those and other challenges be identified in each national onchocerciasis elimination program, and appropriate measures be implemented to accelerate the elimination of onchocerciasis. Some recommendations to overcome the aforementioned challenges and suggested actions to accelerate the elimination of onchocerciasis by 2025 include: a need for complete disease elimination mapping, a need for collaborative elimination activities between national programs, a need for a different drug distribution approach in conflict-affected areas, a need for routine monitoring and evaluation of MDA programs, a need for implementing ATSs in areas with elimination anticipated beyond 2025, and a need for strong partnerships and continued funding.

## Additional file

Additional file 1: Multilingual abstracts in the five official working languages of the United Nations. (PDF 374 kb)

## Data Availability

Not applicable

## Abbreviations

APOC: African Programme for Onchocerciasis Control
ATS: Alternative treatment strategy
CAR: Central African Republic
CDD: Community drug distributor
CDTI: Community-directed treatment with ivermectin
DRC: Democratic Republic of the Congo
ESPEN: Expanded Special Project for Elimination of Neglected Tropical Diseases
MDA: Mass drug administration
NGO: Non-governmental organization
NTD: Neglected tropical disease
OCP: Onchocerciasis Control Programme in West Africa
PCR: Polymerase chain reaction
PTS: Post-treatment surveillance
WHO: World Health Organization
