# Supplementary material for: Reaching the last mile: main challenges relating to and recommendations to accelerate onchocerciasis elimination in Africa
Source: Infect Dis Poverty. 2019 Jul 4;8:60. doi: 10.1186/s40249-019-0567-z (PMC6609392; doi:10.1186/s40249-019-0567-z)
Supplement: Supplementary file 1 — Multilingual abstracts in the five official working languages of the United Nations. (PDF 374 kb) [file 40249_2019_567_MOESM1_ESM.pdf]

Translation of the abstract into the five official working languages of the United Nations

الوصول إلى الميل الأخير: التحديات الرئيسية المتعلقة بالقضاء علي داء كلابية المرض في افريقيا والتوصيات بالتعجيل بها

Gebremedhin Gebrezgabiher, Zeleke Mekonnen, Delenasaw Yewhalaw and Asrat Hailu

مجرده

بالنواقل منقول خطير مهمل استوائي مرض هو ، الخيطية الدودة فصيله عن الناجم ، النهري العمي داء **:الخلفية** ، الذلفاء جنس من الأسود الذباب من لدغه خلال من وينتقل .والاقتصادي الاجتماعي والاهتمام العامة الصحة من في المرض كلابية لداء العالمية والأعباء المخاطر إجمالي من المائة في 99 .والعين الجلدية آلافات في ويتجلى بحلول المرض كلابية داء علي بالقضاء المتعلقة الرئيسية التحديات النطاق لتحديد الاستعراض هذا ويبحث .افريقيا للعثور علي الامراض .الامراض علي بالقضاء والتعجيل التحديات علي للتغلب توصيات ويقترح ، افريقيا في 2020-2025 عام و PubMed بيانات قواعد في البحث تم ، النظراء مراجعه مجلات في المنشورة الصلة ذات المقالات علي Google للباحثين.

في عليه والقضاء الشوك كلابية داء علي للسيطرة نفذت التي الصارمة الاقليميه التدخلات كانت **:الرئيسي النص** عامه صحية مشكله حاليا ليست وهي ؛ المرض عبء علي السيطرة في فعاله افريقيا في الماضية الاربعه العقود .التوطن المفرطة المحليات بعض في الطفيلي انتقال توقف ، الخصوص وجه وعلي .الموبوءة المناطق معظم في في 2020ب الامراض علي الكامل القضاء إلى السيطرة من العامة السياسة في تحول حدث ، الاخيره الاونه وفي توجيهيه مبادئ البشرية الصحة منظمه نشرت وقد .المتوطنة الافريقيه البلدان غالبيه في 2025وب مختاره بلدان نقحت ، ولذلك .الإنسان كلابية داء علي والقضاء العدوى انقطاع من والتحقق للمخدرات الشاملة الاداره لوقف من والتحقق MDA لوقف الادله إلى يستند قرار مع تمشيا الامراض علي للقضاء هدفا ووضعت ، خططها البلدان الوطنية الرئيسية خططها في ودمجها ، عليها القضاء NTDs.

في نستعرضه الذي والتحدي .افريقيا في المرض كلابية داء علي بالقضاء تتعلق تحديات هناك تزال لا ، ذلك ومع ظهور واحتمال ، اللوياسيس كلابية داء مع والتعايش ، الإرسال مناطق لجميع المكتمل غير المسح :هو الورقة هذه البرنامج وتنفيذ ، المدنية الاضطرابات ، والصراع ، الحدود علي للقضاء المنسقة غير والجهود ، ايفرمكتين مقاومه بالقضاء والتعجيل التحديات علي للتغلب توصيات أيضا الورقة هذه وتقترح .والمالية التقنية والتحديات ، الأمثل دون القضاء أنشطه إلى والحاجة ، الامراض علي للقضاء كامله خرائط رسم إلى الحاجة :هي وهذه .الامراض علي والحاجة ، بالنزاع المتاثره المناطق في المخدرات لتوزيع مختلف نهج إلى والحاجة ، الوطنية البرامج بين التعاوني المناطق في (ATSs) البديلة العلاج استراتيجيات تنفيذ إلى والحاجة ، MDA برامج الروتينيين والتقييم الرصد إلى المستمر والتمويل قويه شراكات إلى والحاجة ، 2025 بعد ما المتوقع القضاء مع.

التام القضاء توخي مع ، وتقدمها تدخلاتها أداء بانتظام وتقيم ترصد ان الوطنية البرامج علي يتعين **:الاستنتاجات** انتقال وقف في المتمثل المستهدف الهدف تعرقل التي العوامل تحديد ويلزم .أراضيها من المرض كلابية داء علي علي القضاء لتسريع ATSs تنفيذ يجب ، ومناسبا ممكنا ذلك كان إذا .تصحيحيه إجراءات اتخاذ وينبغي ، الطفيليات 2025ب الامراض

Translated from English version into Arabic by Hamza Faleh Hamza

最后一英里：非洲消除盘尾丝虫病的主要挑战和建议

Gebremedhin Gebrezgabiher, Zeleke Mekonnen, Delenasaw Yewhalaw, Asrat Hailu

摘要

引言：盘尾丝虫病，又称河盲症，是由旋盘尾丝虫感染引起的重要媒传被忽视热带病。该病通过 *Simulium* 属的黑蝇的叮咬传播，主要表现为真皮和眼部病变。绝大部分全球盘尾丝虫病风险和疾病负担都发生在非洲。本文综述了非洲 2020 年到 2025 年消除盘尾丝虫病的主要挑战，并提出了克服挑战和加速消除的建议。为了查找在同行评审期刊上发表的相关文章，我们搜索了 PubMed 和 Google Scholar 数据库。

正文: 为了控制和消除盘尾丝虫病，过去四十年来，非洲开展的严格区域干预措施，并已有效控制了该病疾病负担。目前，在大多数流行地区，该病已不是重要的公共卫生问题。值得注意的是，一些高度流行去已经阻断了寄生虫的传播。最近，一些国家已将原先的政策，即在 2020 年前（大多数非洲流行国家为 2025 年前）控制该病转变为完全消除。世界卫生组织发布了停止大规模给药（MDA）的指南，并且证实已经中断盘尾丝虫病传播和消除的项目。因此，各国已修改了规划，建立了疾病消除目标，决定终止 MDA，并将其纳入 NTD 国家总体规划中。

但是，非洲消除盘尾丝虫病仍然面临挑战。我们在本文中总结了所有流行区的疾病消除图谱，盘尾丝虫病和洛伊斯病的共同流行区，伊维菌素抗药性出现的可能性，不协调的跨境消除措施，冲突和内乱，项目实施欠佳，以及技术和财务挑战。本文还提出了克服挑战和加速消除疾病的建议，包括需要完成消除疾病图谱，在国家计划之间开展协同消除行动，在受冲突影响地区采用不同的药物分配方法，对 MDA 计划进行常规监测和评估，在计划 2025 年以后消除的地区采取替代治疗策略（ATSs），并需要强有力的伙伴关系和持续的资金支持。

结论：各国家项目需定期监测和评估干预措施的实施和进展，并以彻底消除其区域内的盘尾丝虫病作为目标。需要确定实现寄生虫传播阻断目标的阻碍因素，并采取补救措施。如若可行，需使用 ATSs 来加速实现 2025 年消除盘尾丝虫病。

Translated from English version into Chinese by Cong-Shan Liu, edited by Jin Chen, through

CHINESE CENTER FOR DISEASE CONTROL AND PREVENTION
NATIONAL INSTITUTE OF PARASITIC DISEASES

La dernière ligne droite : principales difficultés liées à l’élimination de l’onchocercose en Afrique et recommandations pour l’accélérer

Gebremedhin Gebrezgabiher, Zeleke Mekonnen, Delenasaw Yewhalaw et Asrat Hailu

Résumé

Contexte : L’onchocercose ou cécité des rivières, causée par le ver filaire *Onchocerca volvulus*, est une grave maladie tropicale vectorielle négligée (MTN), préoccupante du point de vue de la santé publique mais aussi par son poids socioéconomique. Transmise par la piqûre des mouches noires du genre *Simulium*, elle se manifeste par des lésions cutanées et oculaires. L’Afrique supporte 99 % de la totalité du fardeau et du risque mondiaux de l’onchocercose.Le présent examen de la portée étudie les principales difficultés liées à l’élimination de l’onchocercose en Afrique à l'horizon 2020-2025 et propose des recommandations pour surmonter les difficultés et accélérer l’élimination de la maladie. Nous avons recherché des articles pertinents, publiés dans des revues à comité de lecture, dans les bases de données PubMed et Google Scholar.

Texte principal : Les interventions régionales rigoureuses déployées au cours des quarante dernières années en Afrique dans le but de lutter contre l’onchocercose et de l'éliminer ont réussi à réduire le fardeau de cette maladie qui n’est plus actuellement un problème de santé publique dans la plupart des zones d’endémie. La transmission du parasite est interrompue, en particulier, dans certaines régions d’hyperendémie. L’approche est récemment passée de la lutte à l’élimination totale de la maladie, d’ici à 2020 dans certains pays, et 2025 dans la plupart des pays africains où l’onchocercose est endémique. L’OMS a publié des recommandations pour que la distribution massive de médicaments soit stoppée et que l’interruption de la transmission et l’élimination de l’onchocercose humaine soient vérifiées. Les pays ont donc revu leurs programmes, fixé un objectif d’élimination de la maladie correspondant à une décision, fondée sur les preuves, d’arrêter la distribution massive de médicaments et de vérifier l’élimination, et ont intégré cet objectif dans leurs plans directeurs nationaux relatifs aux MTN.

L’élimination de l’onchocercose en Afrique continue néanmoins de poser des difficultés. Celles que nous examinons dans le présent article concernent la cartographie incomplète de l’élimination dans toutes les zones de transmission, la co-endémicité de l’onchocercose et de la filariose à loa loa, l’émergence possible d’une résistance à l’ivermectine, l’absence de coordination des efforts d’élimination transfrontaliers, les conflits armés et l’agitation civile, l'insuffisance de la mise en oeuvre des programmes et les difficultés techniques et financières. Le présent article propose également des recommandations pour surmonter les difficultés et accélérer l’élimination de la maladie, à savoir : le besoin d'établir une cartographie complète de l’élimination de la maladie ; le besoin, pour les programmes nationaux, de collaborer sur des activités d'élimination ; le besoin d'adopter une approche différente de distribution des médicaments dans les zones de conflit ; le besoin de contrôler et d'évaluer régulièrement les programmes de distribution massive de médicaments ; le besoin de déployer des stratégies thérapeutiques différentes dans les zones où l’élimination est prévue pour une date postérieure à 2025 ; un besoin de partenariats forts et de financements continus.

Conclusions : Les programmes nationaux doivent contrôler et évaluer régulièrement les résultats et la progression de leurs interventions, tout en planifiant l’élimination totale de l’onchocercose sur leur territoire. Les facteurs qui empêchent d’atteindre l'objectif d’interruption de la transmission du parasite doivent être identifiés et des actions entreprises pour y remédier. Dans la mesure du possible, des stratégies thérapeutiques différentes doivent être déployées afin d’accélérer l’élimination de la maladie d’ici 2025.

Translated from English version into French by Suzanne Assenat, Revised by Cendrine Strevens, through

TRANSLATORS
WITHOUT BORDERS

Дойти до конца пути: основные проблемы и рекомендации, связанные с ускорением ликвидации онхоцерхоза в Африке

Гебремедхин Гебрезгабихер, Зэлекэ Маконнен, Деленасав Евхалав и Асрат Хайлу

Реферат
Предпосылки: онхоцеркоз (речная слепота), вызываемый филярийными нематодами *Onchocerca volvulus*, является серьезной трансмиссивной забытой тропической болезнью (ЗТБ) и проблемой для системы здравоохранения и социоэкономики. Болезнь передается через укусы черных мошек семейства*Simulium* и характеризуется поражением кожи и глаз. Девяносто девять процентов от общего глобального риска и бремени онхоцеркоза приходятся на Африку. В предварительном обзоре изучаются ключевые проблемы, связанные с ликвидацией онхоцеркоза к 2020-2025 гг. в Африке и представлены рекомендации по преодолению этих проблем и ускорению ликвидации болезни. Дляпоиска релевантных публикаций в научных рецензируемых журналах был проведен поиск по базам данных PubMed и Google Scholar.
Основной текст. Строгие региональные меры, предпринимаемые для борьбы и ликвидации онхоцеркоза в Африке в последние четыре десятилетия, были эффективными для установления контроля над бременем болезни; на данный момент она не является проблемой здравоохранения в наиболее эндемичных районах. Примечательно то, что передача паразита прервана в некоторых гиперэндемичных местностях. С недавних пор в политике произошел переход от борьбы к полной ликвидации болезни к 2020 году в избранных странах и к 2025 году — в большинстве эндемичных африканских стран. ВОЗ опубликовала руководства по прекращению массового введения лекарственных препаратов и обеспечению прерывания передачи и ликвидации онхоцеркоза у людей. Поэтому власти стран пересмотрели свои планы, определив цель — ликвидировать болезнь вместе с основанном на доказательствах решении прекратить массовое введение лекарственных препаратов и верифицировать ликвидацию болезни, и включили ее в свои национальные генеральные планы по ЗТБ.
Несмотря на это, проблемы, связанные с ликвидацией онхоцеркоза в Африке, остаются. Проблемы, рассматриваемые в данной работе, таковы: недостаточное картирование ликвидации всех зон передачи, соэндемичных и по онхоцеркозу, и по лоаозу, вероятность возникновения устойчивости к ивермектину, нескоординированность попыток ликвидации чрезграничного переноса, конфликт и гражданские беспорядки, неоптимальная реализация программы и технические и финансовые проблемы. В данном документе также представлены рекомендации по преодолению этих проблем и ускорению ликвидации болезни. К ним относятся следующие: необходимость полного картирования ликвидации болезни, необходимость согласования действий по ликвидации между национальными программами, необходимость иного подхода к распространению лекарственных препаратов в затронутых конфликтом районах, необходимость проведения рутинного мониторинга и оценки программ прекращения массового введения лекарственных препаратов, необходимость внедрения альтернативных стратегий лечения в областях, где ликвидация болезни ожидается после 2025 г., и необходимость крепкого партнерства и продолжения финансирования.
Выводы: в рамках национальных программ необходимо регулярно проводить мониторинг и оценку хода таких вмешательств в свете полной ликвидации онхоцеркоза на соответствующей территории. Необходимо выделить факторы, препятствующие

достижению намеченной цели прерывания передачи паразитаи принять меры к решению этой проблемы. При возможности и целесообразности для ускорения процесса ликвидации болезни к 2025 г. необходимо внедрить альтернативные стратегии лечения.

Translated from English version into Russian by Veronika Demeshchik, Revised by Alexander Somin, through

TRANSLATORS WITHOUT BORDERS

Recorrer el último kilómetro: principales desafíos relacionados con la aceleración de la eliminación de la oncocercosis en África y recomendaciones para ello

Gebremedhin Gebrezgabiher, Zeleke Mekonnen, Delenasaw Yewhalaw y Asrat Hailu

Resumen

Antecedentes: la oncocercocis (conocida como la ceguera de los ríos), causada por el nematodo filiar *Onchocerca volvulus*, es una grave enfermedad tropical desatendida (ETD) transmitida por vectores que constituye un problema socioeconómico y de salud pública. Se transmite a través de la picadura de moscas negras del género *Simulium*, y se manifiesta mediante lesiones cutáneas y oculares. El noventa y nueve por ciento de la incidencia y el riesgo global total de la oncorcercosis se encuentra en África. Esta revisión del alcance examina los principales desafíos relacionados con la eliminación de la oncocercosis para 2020-2025 en África, y propone recomendaciones para superar los desafíos y acelerar la eliminación de la enfermedad. Se llevó a cabo una búsqueda en las bases de datos de PubMed y Google Académico para encontrar artículos relevantes publicados en revistas arbitradas.
Texto principal: las rigurosas intervenciones regionales efectuadas durante las últimas cuatro décadas en África para controlar y eliminar la oncocercosis han controlado la incidencia de la enfermedad de manera eficaz. Actualmente no representa un problema de salud pública en la mayoría de las zonas endémicas. En especial, la transmisión del parásito se ha interrumpido en algunas localidades hiperendémicas. Recientemente, ha ocurrido un cambio de orientación en las políticas que vira del control a la eliminación total de la enfermedad para el año 2020 en países seleccionados y para el año 2025 en la mayoría de los países endémicos africanos. La OMS ha publicado directrices para suspender la administración masiva de medicamentos (AMM) y para verificar la interrupción de la transmisión y la eliminación de la oncocercosis humana. Por lo tanto, los países han revisado sus planes y establecido una meta para la eliminación de la enfermedad coherente con una decisión basada en pruebas de suspender la AMM y verificar la eliminación, y la han incorporado a sus planes maestros a nivel nacional contra las ETD.
Sin embargo, algunos desafíos relacionados con la eliminación de la oncocercosis en África aún persisten. Los desafíos que reseñamos en este artículo son: el mapeo incompleto de la eliminación en todas las zonas de transmisión, la coendemicidad de la oncocercosis y la loaisis, la posible aparición de una resistencia a la ivermectina, los esfuerzos transfronterizos no coordinados para la

eliminación de la enfermedad, los conflictos y disturbios civiles, la implementación deficiente del programa y los desafíos técnicos y financieros. Este artículo también propone recomendaciones para superar los desafíos y acelerar la eliminación de la enfermedad. Estas son: la necesidad de un mapeo completo de la eliminación de la enfermedad, la necesidad de actividades conjuntas de eliminación entre programas nacionales, la necesidad de un enfoque de distribución de medicamentos distinto en zonas afectadas por conflictos, la necesidad de supervisión y evaluación rutinaria de programas de AMM, la necesidad de implementar estrategias de tratamiento alternativas (ATS, por sus siglas en inglés) en zonas donde se anticipa la eliminación después del año 2025 y la necesidad de asociaciones sólidas y financiación continua.
Conclusiones: los programas nacionales deben supervisar y evaluar periódicamente el desempeño y el progreso de sus intervenciones, mientras contemplan la eliminación total de la oncocercosis en su territorio. Se deben identificar los factores que obstaculizan la consecución del objetivo pautado de interrumpir la transmisión del parásito y se deben tomar medidas correctivas al respecto. Cuando sea posible y apropiado, deben implementarse ATS para acelerar la eliminación de la enfermedad para el año 2025.

Translated from English version into Spanish by Maria Gracia Zavarse, Revised by Mayra León, through

TRANSLATORS
WITHOUT BORDERS
